# Protective and Therapeutic Efficacy of Hesperidin versus Cisplatin against Ehrlich Ascites Carcinoma-Induced Renal Damage in Mice

**DOI:** 10.3390/ph15030294

**Published:** 2022-02-28

**Authors:** Nahed Saleh, Tamer Allam, Reda M. S. Korany, Abdelfattah M. Abdelfattah, Ahmed M. Omran, Mabrouk Attia Abd Eldaim, Aziza M. Hassan, Nermeen Borai El-Borai

**Affiliations:** 1 Department of Clinical Pathology, Faculty of Veterinary Medicine, University of Sadat City, Sadat City 32897, Menoufia, Egypt; nahed.thabet@vet.usc.edu.eg (N.S.); tamer.salah@vet.usc.edu.eg (T.A.); abdelfattah.mohamed@vet.usc.edu.eg (A.M.A.); ahmed.omran@vet.usc.edu.eg (A.M.O.); 2 Department of Pathology, Faculty of Veterinary Medicine, Cairo University, Giza 12211, Egypt; reda_pathology@cu.edu.eg; 3 Department of Biochemistry and Chemistry of Nutrition, Faculty of Veterinary Medicine, Menoufia University, Sheben El-Kom 32511, Egypt; 4 Department of Biotechnology, Collage of Science, Taif University, P.O. Box 11099, Taif 21944, Saudi Arabia; a.hasn@tu.edu.sa; 5 Department of Forensic Medicine & Toxicology, Faculty of Veterinary Medicine, University of Sadat City, Sadat City 32897, Menoufia, Egypt

**Keywords:** Ehrlich ascites carcinoma, hesperidin, cisplatin, carcinoembryonic antigen, Ki-67, caspase-3

## Abstract

This study evaluates the antitumor efficacy of hesperidin (Hesp) versus cisplatin (Cis) in Ehrlich ascites carcinoma (EAC)-bearing mice, as well as its protective effect against Cis-triggered nephrotoxicity. Seventy female mice were allocated into control, Hesp, EAC, Hesp-protected, Hesp-treated, Cis-treated, and Cis+Hesp-treated groups. The inoculation of mice with EAC cells significantly reduced the mean survival time, while significantly increased the body weight, abdominal circumference, ascitic fluid volume, viable tumor cell count, and serum carcinoembryonic antigen, urea and creatinine levels, besides various hematological changes. Additionally, kidney tissue of EAC-bearing mice showed a significant increase in the malondialdehyde level, significant decreases in the reduced glutathione content and catalase activity, marked pathological alterations, and a strong Ki-67 expression with a weak caspase-3 expression in neoplastic cells infiltrating the renal capsule. Conversely, the administration of Hesp and/or Cis to the EAC-bearing mice induced, to various degrees, antitumor responses and alleviated the cytotoxic effects of EAC. In addition to the potent antitumor effect of the concomitant administration of Hesp and Cis, Hesp minimized the renal adverse side effects of Cis. In conclusion, Hesp may open new avenues for safe and effective cancer therapy and could be valuable for enhancing the antitumor potency and minimizing the renal adverse side effects of chemotherapeutic drugs.

## 1. Introduction

Ehrlich ascites carcinoma (EAC) is a spontaneous mammary adenocarcinoma that is well-established in the experimental model of cancers [1]. Resembling human tumors, EAC is characterized by a rapid and undifferentiating proliferation, shorter life span, and 100% malignancy [2]. In addition, EAC is highly sensitive to chemotherapy and, hence, it is widely used in cancer, antineoplastic, and chemotherapeutic studies [2]. Despite the wide use of chemotherapeutic drugs for cancer treatment, unfortunately, most of the currently used drugs are specifically nonselective for neoplastic cells, resulting in numerous forms of organ damage [3].

Cis-diamminedichloroplatinum-(II) or cisplatin (Cis) is the most frequently used and one of the few exceptionally successful chemotherapeutic drugs with a broader efficacy in the treatment of a wide spectrum of malignancies, which acts via activating a variety of signaling pathways in cancer cells [4]. However, over recent decades, the chemotherapeutic efficacy of Cis has been limited by the subsequent drug resistance and its various adverse side effects, including nephrotoxicity, neurotoxicity, hepatotoxicity, ototoxicity, gastric disorders, and myelosuppression [4,5]. Thus, there are growing concerns about the development of effective and side-effect-free anticancer drugs. Nowadays, chemoprevention is a promising approach for cancer management by using synthetic and/or natural agents for the prevention of cancer progression in humans [6].

Medicinal plants are considered one of the most effective and safe therapeutic agents and a major source of phenols and flavonoids that exhibit health-promoting effects and exert a pivotal role as an alternative medicine for the treatment of human diseases and management of cancer [7].

Hesperidin (Hesp) is a natural bioflavonoid that is abundantly found in the peel and membranous parts of various citrus fruits, such as oranges, lemons, mandarins, and grapefruit [8]. Interestingly, Hesp has gained great attention in the treatment of various oxidative stress-mediated disorders such as cardiovascular and hepatorenal diseases, neurological disorders, diabetes, aging, and cancer [8,9,10]. In addition, Hesp has potent antiatherosclerotic, anti-inflammatory, antioxidant, antiallergic and antimicrobial properties [11]. Recently, many researchers have emphasized how Hesp can modulate the hallmarks of cancer through downregulating pro-inflammatory mediators and enzymes, improving the antioxidant defense system, and inhibiting cancer cell proliferation by increasing apoptosis in cancer cells [12].

Set against this background, the current study is performed, firstly, to compare the antitumor efficacy of Hesp versus Cis in EAC-bearing mice, and also to evaluate the possible role of the concomitant treatment of Hesp in enhancing the antitumor potency of Cis, as well as its protective effect against Cis-triggered nephrotoxicity.

## 2. Results

### 2.1. Hesperidin and/or Cisplatin Improved the General Health Condition of EAC-Bearing Mice

Along the experimental period, no apparent symptoms were observed in either the control or Hesp groups. However, the gradual swelling of the abdomen, dullness, depression, loss of activity, and decreased appetite were the main clinical manifestations in EAC-bearing mice. Meanwhile, the administration of Hesp and/or Cis to EAC-bearing mice exhibited less severe symptoms. A marked improvement was observed in the Hesp-protected and Cis+Hesp-treated groups.

### 2.2. Hesperidin and/or Cisplatin Increased MST and ILS% in EAC-Bearing Mice

As illustrated in Table 1, EAC-bearing mice showed a remarkable decrease in the MST. However, the administration of Hesp and/or Cis to EAC-bearing mice resulted in notable increases in the MST and ILS%, with the highest values observed in the Hesp-protected and Cis+Hesp-treated groups.

### 2.3. Hesperidin and/or Cisplatin Alleviated Tumor Growth Responses in EAC-Bearing Mice

Data in Table 1 showed no significant (*p* < 0.05) differences in the body weight and abdominal circumference values of control and Hesp groups. Concerning the values of the control group, significant increases in the body weight and abdominal circumference values were recorded in the EAC-bearing mice. Compared to the EAC-bearing mice, the protection of EAC-bearing mice with Hesp or the treatment with Hesp and/or Cis showed significant decreases in the body weight and abdominal circumference values, and the body weight and abdominal circumference of the Hesp-protected and Cis+Hesp-treated groups were restored to the normal control values.

The inoculation of mice with EAC significantly (*p* < 0.05) increased the ascetic fluid volume and viable EAC cells count, while the nonviable EAC cells count was significantly decreased. Regarding the mean values of the EAC group, the administration of Hesp and/or Cis induced significant decreases in the corresponding values. Importantly, the highest improvements were recorded in the Hesp-protected and Cis+Hesp-treated groups followed by the Cis-treated and Hesp-treated groups (Table 1).

### 2.4. Hesperidin and/or Cisplatin Decreased Serum CEA Level in EAC-Bearing Mice

The serum level of CEA showed no significant (*p* < 0.05) differences between the control and Hesp groups. A significant increase in the serum CEA level was observed in the EAC-bearing mice, compared to the control group. However, the protection of EAC-bearing mice with Hesp or their treatment with Hesp and/or Cis showed a significant decrease in the corresponding values, compared with the EAC-bearing mice, and restored them to the normal control values in the Hesp-protected and Cis+Hesp-treated groups (Table 1).

### 2.5. Hesperidin and/or Cisplatin Modulated the Hematological Changes in EAC-Bearing Mice

The effect of administration of Hesp and/or Cis of the EAC-bearing mice on erythrogram indices is presented in Table 2. No significant (*p* < 0.05) differences were recorded in the mean values of RBCs, Hb, PCV, MCV, MCH, and MCHC between the control and Hesp groups. Conversely, EAC-bearing mice exhibited significant decreases in the mean values of RBCs, Hb, and PCV, along with a significant increase in the mean value of MCV without any changes in the mean values of MCH and MCHC, compared to the control values. Compared to the mean values of the EAC group, the Hesp-protected and Cis+Hesp-treated groups showed significant improvements and restored erythrogram indices to the normal control values. On the other hand, no significant changes were recorded in both the Hesp-protected and Cis+Hesp-treated groups, compared to the EAC group (Table 2).

Referring to the changes in the leukogram and blood platelets, data presented in Table 3 showed no significant differences between the control and Hesp groups. However, the inoculation of mice with EAC exhibited significant increases in the counts of TWBCs, granulocytes, and monocytes, and a significant decrease in the platelets count, compared to those of the control group. The treatment of EAC-bearing mice with Hesp showed a significant improvement only in the granulocytes count, compared to the EAC group. However, significant improvements were observed in the TWBC, granulocyte, monocyte, and platelet counts of the other groups, compared to those of the EAC group.

### 2.6. Hesperidin and/or Cisplatin Decreased Serum Urea and Creatinine Levels of EAC-Bearing Mice

**Figure 1:**
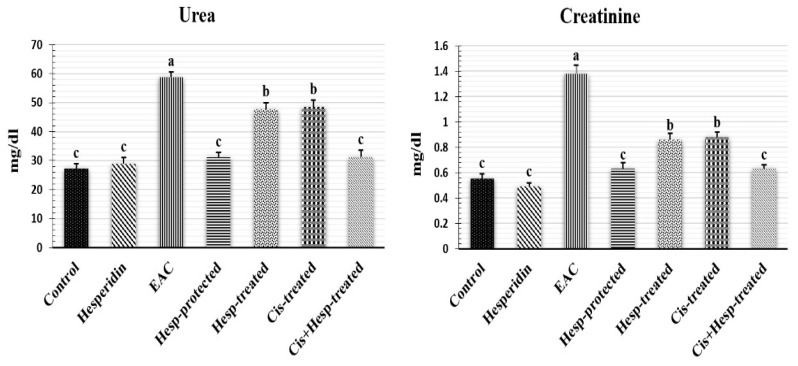
Effect of hesperidin and/or cisplatin on serum urea and creatinine levels of EAC-bearing mice (*n*= 7). Different letters (a, b, c) indicate significant differences at *p* < 0.05. EAC: Ehrlich ascites carcinoma; Hesp: hesperidin; Cis: cisplatin.

As displayed in Figure 1, no significant (*p* < 0.05) differences were recorded in the mean values of serum urea and creatinine levels between the control and Hesp groups. Conversely, significant elevations in the levels of urea and creatinine were recorded in the EAC-bearing mice in comparison with the control mice. Regarding the mean values of the EAC group, the administration of Hesp and/or Cis induced significant decreases in the corresponding values with restoring the normal control values in the Hesp-protected and Cis+Hesp-treated groups.

### 2.7. Hesperidin and/or Cisplatin Improved the Renal Oxidant/Antioxidant Status in EAC-Bearing Mice

**Figure 2:**
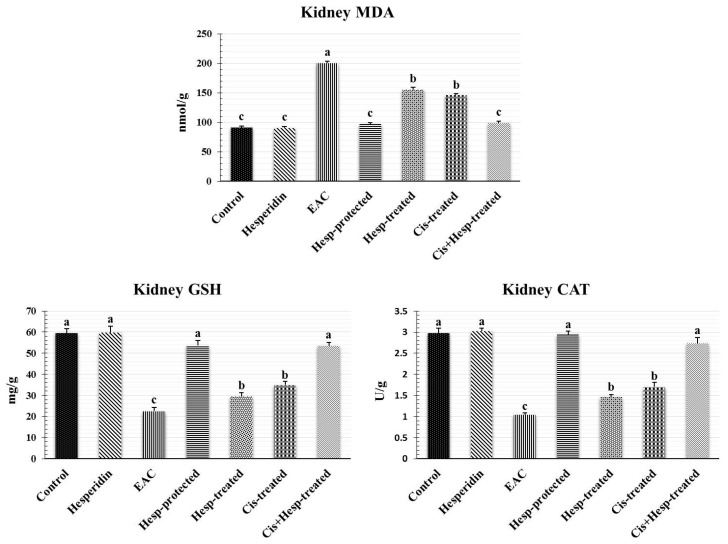
Effect of hesperidin and/or cisplatin on renal oxidant/antioxidant status EAC-bearing mice (*n* = 7). Different letters (a, b, c) indicate significant differences at *p* < 0.05. EAC: Ehrlich ascites carcinoma; Hesp: hesperidin; Cis: cisplatin; MDA: malondialdehyde; GSH: reduced glutathione; CAT: catalase.

Results shown in Figure 2 demonstrated that the administration of Hesp did not induce any significant (*p* < 0.05) effect on the mean values of renal oxidant/antioxidant biomarkers, compared to those of the control group. However, the inoculation of EAC in mice induced a significant elevation in the renal MDA level, together with significant reductions in the GSH content and CAT activity, compared to the corresponding control values. Interestingly, the administration of Hesp and/or Cis significantly ameliorated the EAC-induced alterations in the oxidant/antioxidant biomarkers, compared to the corresponding values of the EAC group, with reinstating their normal control values in the Hesp-protected and Cis+Hesp-treated groups.

### 2.8. Hesperidin and/or Cisplatin Improved the Renal Histoarchitecture in EAC-Bearing Mice

**Figure 3:**
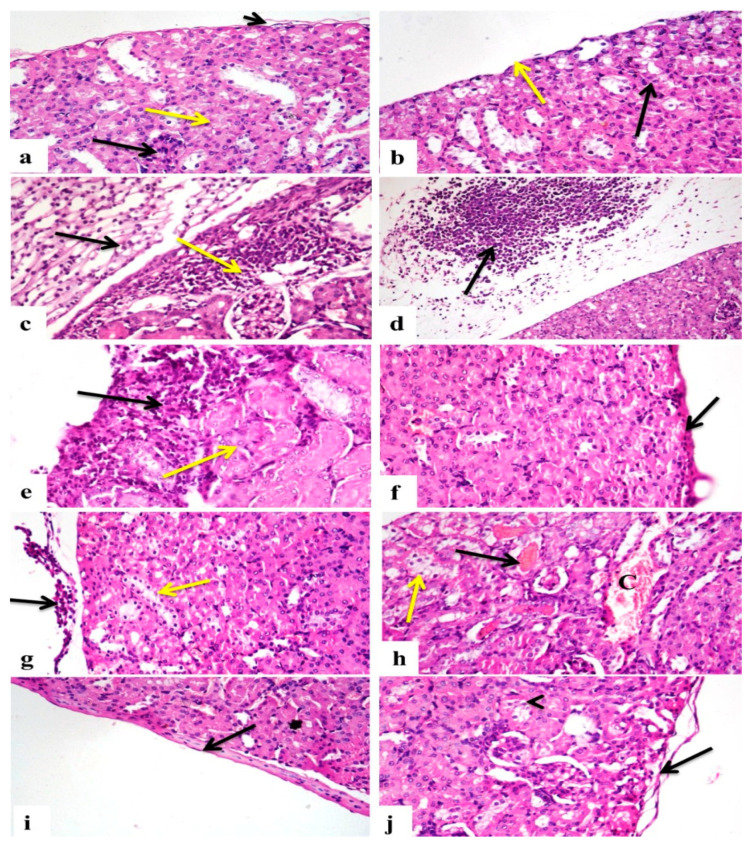
Photomicrographs of histopathological alterations in kidney sections of different groups (H&E stain ×400). (**a**) Control and (**b**) Hesp groups showing normal structure of renal corpuscles (black arrow), tubules (yellow arrow), and capsule (short arrow). (**c**) EAC group showing mononuclear inflammatory cells (yellow arrow), edema, and inflammatory cells in capsule (black arrow). (**d**) EAC group showing infiltration of cancer cells in capsule (arrow). (**e**) Hesp-protected group showing mononuclear inflammatory cells (black arrow) with normal tubules (yellow arrow). (**f**) Hesp-protected group showing capsule free of neoplastic cells (arrow). (**g**) Hesp-treated group showing infiltration of capsule with cancer cells (black arrow) with vacuolar degeneration of tubules (yellow arrow). (**h**) Cis-treated group showing necrosis of tubular lining epithelium with presence of hemoglobin cast (black arrow), vacuolar degeneration of tubule (yellow arrow), and congestion of interstitial blood vessels (C). (**i**) Cis-treated group showing capsule free of cancer cells (arrow). (**j**) Cis+Hesp-treated group showing free capsule of cancer cells (arrow) with mild vacuolar degeneration of tubules (arrowhead).

The histopathological alterations in kidneys of different treated groups are presented in Table 4 and Figure 3. Kidney sections of the control (Figure 3a) and Hesp groups (Figure 3b) appeared normal with no definite histopathological changes. Kidney sections of the EAC group showed congestion in the glomerular tuft and interstitial blood vessels, interstitial tissue was infiltrated with mononuclear inflammatory cells, renal tubules revealed the presence of vacuolar degeneration and necrosis, and the renal capsule showed an increase in its thickness with an edema and mononuclear inflammatory cells infiltration (Figure 3c), the renal capsule also having been heavily infiltrated with neoplastic cells that were characterized by nuclear hyperchromasia and increased the nuclear–cytoplasmic ratio (Figure 3d). Kidney sections of the Hesp-protected group revealed a few interstitial mononuclear inflammatory cell infiltrations, with a normal renal tubular lining epithelium (Figure 3e) or even regenerating nodules, the interstitial blood vessels were moderately congested, and the renal capsule was normal with a few or no infiltrating cancer cells (Figure 3f). In contrary, the Hesp-treated group showed capsular infiltrations with cancer cells with the degeneration of the tubular lining epithelium (Figure 3g), interstitial blood vessels and glomerular tuft were congested, there were mononuclear inflammatory cell infiltrations in interstitial tissue, some glomeruli showed necrosis of their tuft, and some renal tubules were cystically dilated and necrotized with the presence of protein casts inside their lumen. Additionally, the Cis-treated group revealed mononuclear inflammatory cell infiltrations in interstitial tissue with the congestion of interstitial blood vessels, some renal tubules were necrotized with the presence of hemoglobin casts, some were degenerated (Figure 3h), others were cystically dilated, and the renal capsule showed the presence of a few or was even free of neoplastic cells (Figure 3i). On the other hand, the Cis+Hesp-treated group showed a few interstitial mononuclear inflammatory cell infiltrations with mild congestion of interstitial blood vessels, some renal tubules showed mild vacuolar degeneration, and the renal capsule revealed a few or no infiltrating cancer cells (Figure 3j).

### 2.9. Hesperidin and/or Cisplatin Upregulated Ki-67 and Downregulated Caspase-3 Proteins Expressions of the Neoplastic Cells in Renal Capsule of EAC-Bearing Mice

**Figure 4:**
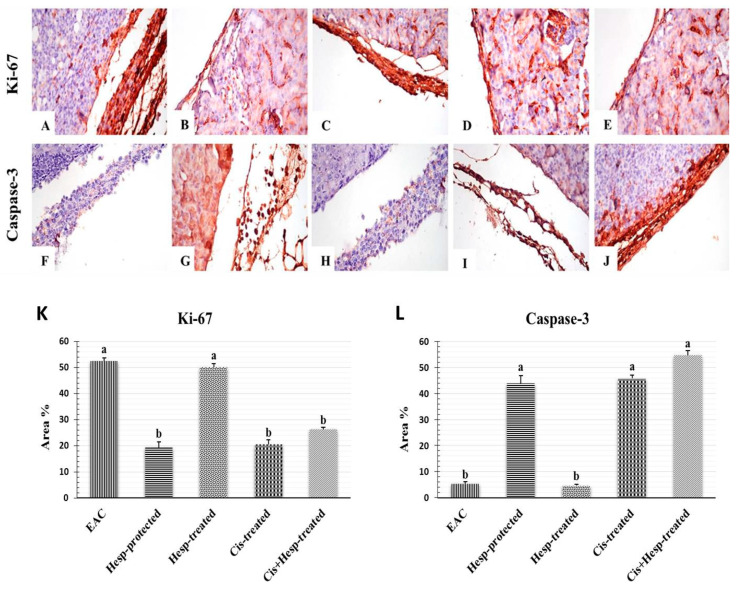
Photomicrographs of immuno-histochemical reactivity of Ki-67 (**A**–**E**) and caspase-3 (**F**–**J**) in renal capsule of different groups (×400). (**A**,**F**) EAC group showing strong expression of Ki-67 and no immune-reactive cells, with caspase-3 in neoplastic cells infiltrating renal capsule. (**B**,**G**) Hesp-protected group showing weak expression of Ki-67 and strong expression of caspase-3 in few neoplastic cells infiltrating renal capsule. (**C**,**H**) Hesp-treated group showing strong positive expression of Ki-67 and no immune-reactive cells with caspase-3 in neoplastic cells in renal capsule. (**D**,**I**) Cis-treated group showing weak expression of Ki-67 and strong expression of caspase-3 in neoplastic cells infiltrating renal capsule. (**E**,**J**) Cis+Hesp-treated group showing weak expression of Ki-67 and strong expression of caspase-3 in renal capsule. (**K**,**L**) Immunostaining expression of Ki-67 and caspase-3 area % in neoplastic cells of different treated groups; the values are expressed as means ± SE (*n* = 5). Different letters (a, b) indicate significant differences at *p* < 0.05. EAC: Ehrlich ascites carcinoma; Hesp: hesperidin; Cis: cisplatin.

Figure 4 presents the immune reactivity for Ki-67 and caspase-3 in the neoplastic cells in the renal capsule of different treated groups. The Immunostaining of Ki-67 in the EAC group revealed a strong expression of Ki-67 in neoplastic cells infiltrating the renal capsule, and a weak or no immune-reactive neoplastic cells with caspase-3 (Figure 4A,F), while the Hesp-protected group showed a weak expression of Ki-67 in neoplastic cells infiltrating the renal capsule and a strong expression of caspase-3 in neoplastic cells (Figure 4B,G). The Hesp-treated group showed a strong positive expression of Ki-67 in neoplastic cells, and a weak or no immune-reactive neoplastic cells with caspase-3 in the renal capsule (Figure 4C,H). Furthermore, the Cis-treated group showed a weak expression of Ki-67 in neoplastic cells and a strong expression of caspase-3 in neoplastic cells (Figure 4D,I). On the other hand, the Cis+Hesp-treated group showed a weak expression of Ki-67 with a strong expression of caspase-3 in the renal capsule (Figure 4E,J). The immunostaining expression of Ki-67 and caspase-3 area % in neoplastic cells of different treated groups was illustrated (Figure 4K,L).

## 3. Discussion

Cancer is considered the second leading cause of death worldwide [13]. Although chemotherapeutic drugs are effective against many types of cancer, they are limited by various adverse side effects and complications [14]. Due to the continuous increase in the global incidence of malignancies, great attention has been paid to exploring safe and efficient strategies for cancer treatment. Nowadays, many natural products from herbs, vegetables, plant extracts, and fruits have been found to have chemoprotective properties against carcinogenesis [15].

Herein, EAC-bearing mice showed a notable reduction in the mean survival time and marked increases in the final body weight, abdominal circumference, ascitic fluid volume, and viable tumor cell count. These findings were in line with that of Donia et al. [16] and Hashem et al. [17]. High mortalities and a low survival rate may reflect the progressive tumor growth, which was triggered by the inflammatory reaction induced by the EAC with the impairment of blood and lymph return and/or increase in the capillary permeability, leading to a leakage of protein in the abdominal cavity and the accumulation of ascitic fluid [17].

The carcinoembryonic antigen, a well-known tumor marker, is a cell surface-bound glycoprotein that is overexpressed and released by a variety of tumors and exerts an autocrine role in neoplastic cell survival and differentiation [18]. Our results revealed an increase in the serum CEA level in EAC-bearing mice, which were similar to the findings of Hashem et al. [17] and Abd Eldaim et al. [19], who reported an elevation in the serum level of CEA in EAC-bearing mice, indicating the tumor metastasis in different organs, comprising ovarian, pancreatic, gastric, and colorectal tumors [20].

In addition, the current study revealed that EAC induced anemia, manifested by significant reductions in RBCs count, hemoglobin concentration, and PCV value, along with a significant elevation in the MCV value and insignificant changes of MCH and MCHC values, indicating the presence of macrocytic normochromic anemia. Similar findings were reported by Hashem et al. [21], who related these effects to the suppressive effect of EAC on erythropoiesis that could result from iron deficiency, hemolytic or myelopathic conditions [22]. Moreover, the macrocytic normochromic anemia observed in the EAC-bearing mice could be attributed, in part, to the deficiency of folic acid that may be due to EAC-induced thiamine deficiency, which is essential for folic acid metabolism [23,24]. A deficiency of folic acid could also result from intensive EAC cells proliferation, where folic acid is utilized extensively for the production and maintenance of DNA and RNA synthesis for newly grown cells [25]. Consistent with the obtained results, Badr et al. [26] found that the EAC-bearing mice exhibited significant increases in white blood cell, granulocyte, and monocyte counts that may be due to the acute inflammatory response and/or oxidative stress mediated by the proliferation of Ehrlich cells [17]. The recorded thrombocytopenia in the EAC-bearing mice was also recorded by Hashem et al. [21], who attributed that to the suppressive effect of EAC on the bone marrow.

The current study proved the impairment of kidney functions in EAC-bearing mice, which was indicated by the elevated serum urea and creatinine levels. In association with these findings, various vascular, degenerative, and inflammatory pathological changes, along with a heavy infiltration of neoplastic cells were also recorded in the renal tissue of EAC-bearing mice. These findings were in line with those recorded by Donia et al. [16], Hashem et al. [17], and Abd Eldaim et al. [19]. Subsequently, the elevation of kidney function biomarkers could be attributed to the renal damage induced by the tumor metastasis and the infiltration of cancer cells in renal tissue, resulting in the impairment of the glomerular filtration rate and renal tubular reabsorption, and the reduction in urea and creatinine excretion; thus, increasing their blood levels [27].

Oxidative stress is well known to be one of the pivotal triggers for cancer initiation and progression, and it is also implicated as a possible mechanism of EAC-induced renal damage [17]. The kidneys are more susceptible to oxidative damage induced by the excessive generation of ROS, probably due to the plentiful amount of long-chain polyunsaturated fatty acids in the renal lipids [28]. In accordance with our findings, Donia et al. [16] and Medhat et al. [29] recorded renal oxidative damage, evidenced by a significant elevation in the MDA level and reductions in the GSH content and CAT activity. Herein, the oxidative renal damage may be due to the EAC-induced overgeneration of ROS, the precursor for tumor progression that ultimately increases lipid peroxidation and decreases the cellular antioxidants and, subsequently, induces renal tissue damage [17].

Ki-67, a nuclear protein involved in cell proliferation, exists during all active phases of the cell cycle and is commonly used as a marker for the proliferation of cancer cells in EAC-bearing mice [30]. On the other hand, caspase-3 is an apoptotic protein that activates caspase-8, causing DNA damage and cell death [31]. Our immunohistochemical examination revealed a strong expressions of the Ki-67 gene and a weak expression of the caspase-3 gene in neoplastic cells infiltrating the renal capsule of EAC-bearing mice, as previously reported by Hashem et al. [17] and El-Naa et al. [32], indicating that EAC is able to induce proliferation in kidney tissues.

Chemotherapy is the recommended treatment for cancer, which acts mainly by preventing the growth and progression of tumor cells or by destroying them [33]. Cisplatin, a well-known chemotherapeutic drug, is widely used against a variety of malignancies [34]. However, the potent anticancer efficacy of Cis is often hampered by the development of many dose-limiting side effects and by the chemo-resistance of the neoplastic cells [4]. In an attempt to overcome these limitations, the use of Cis in combination with some modulating natural agents is crucial to manage the cytotoxic effects of Cis. The results of the current study showed that the treatment of EAC-bearing mice with Cis induced marked increases in the MST and ILS%, with significant decreases in the final body weight and abdominal circumference values, ascetic fluid volume, and viable EAC cells count, in addition to the significant reduction in the level of the serum tumor marker, CEA. These findings were inconsistent with those recorded by Hashem et al. [17]. Numerous studies recorded the antitumor efficacy of Cis [17,34], which might be related to the interaction between the Cis and DNA molecules, resulting in a superoxide radicals generation that causes the death of cancer cells [35].

Myelosuppression is a major consequent toxic effect of Cis [36]. Herein, Cis induced bone marrow hypocellularity, resulting in remarkable pancytopenia as reported by Hashem et al. [17] and Kuter [37]. The recorded anemia may be attributed to the inhibition of the erythropoietin hormone [38], which correlates with Cis-induced kidney damage as the kidney is the major source of the erythropoietin hormone [39] or may be due to the suppression of bone marrow precursors [40].

The present study showed a significant leukopenia in the Cis-treated group, which may be ascribed to the immunosuppressive effect of Cis [41]. The thrombocytopenia detected in this study may be due to the apoptosis of megakaryocyte progenitors [42] or the depletion of the thrombopoietin hormone [43], which results from kidney damage caused by Cis. From another point of view, the observed pancytopenia may be a consequence of Cis-induced bone marrow depletion [41]. Despite the improvement of renal function in the EAC-bearing mice treated with Cis, the levels of urea and creatinine were still significantly elevated from those of the control values. Similar findings have also been reported in recent studies [21,44,45]. Indeed, nephrotoxicity is a common side effect of Cis medication that may be due to the impairment of renal functions, the back-leakage of the renal tubules, and/or renal tubular obstruction induced by Cis [39,46]. Further, Cis is linked to the damage of the proximal tubular epithelial cells (PTECs) because Cis is reabsorbed by megalin, a glycoprotein that is expressed in PTECs, causing apoptosis and tubular damage [47,48]. Additionally, the impairment of renal functions might be due to the direct oxidative renal damaging effect of Cis on the tubular and glomerular structures via the excessive production of free radicals and the depletion of the renal antioxidant defense system [17], as evidenced in the current study by the observed increase in the renal MDA level and reductions in the GSH content and CAT activity in EAC-bearing mice treated with Cis. These results agreed fully with previous findings of Ali et al. [49] and Longchar and Prasad [34]. In support of these results, the histopathological and immunohistochemical investigations ascertained the biochemical findings, where some vascular, degenerative, and necrotic changes with only a few neoplastic cells were observed in the renal tissue of EAC-bearing mice treated with Cis, along with weak Ki-67 and strong caspase-3 expressions of neoplastic cells infiltrating the renal capsule. Similar findings were also observed by Ali et al. [49], Hashem et al. [17], and Longchar and Prasad [34], indicating the antitumor potency of Cis and its renal damaging adverse side effect.

Hesperidin is the main flavonoid in the peel of various citrus species, which has been proved to possess numerous biological effects, including antioxidant, anti-inflammatory, antiapoptotic, and anticarcinogenic effects [8,9,10,11]. In the present study, the antitumor efficacy of Hesp was evaluated versus Cis in EAC-bearing mice to explore its role in enhancing the antitumor efficacy of Cis and minimizing its adverse side effects. Regarding the cytoprotective and ameliorative potency of Hesp, the current findings revealed that the administration of Hesp alone or in combination with Cis to the EAC-bearing mice induced, to various degrees, antitumor responses, and also alleviated the renal adverse effects of EAC, particularly when Hesp was used as a protective agent against EAC or as a treatment in combination with Cis. The recorded increase in the lifespan of EAC-bearing mice following the administration of Hesp, as well as the reduction in tumor growth responses and the decrease in the CEA level, were considered as valuable indications of the significant antitumor potency of Hesp recently demonstrated by Donia et al. [16] and Khedr and Khalil [50], which could be attributed to its ability to decrease the nutritional supplements and arresting the tumor growth [51]. Recently, Aggarwal et al. [12] suggested that the induction of apoptosis and the cell cycle arrest is among the main mechanisms of Hesp action against neoplastic cells, besides its antioxidant and anti-inflammatory activities. Consistently, Mahmoud [52] proved that Hesp ameliorated the alterations of erythrocytes, leukocytes and their functional indices in diabetic rats. Additionally, Hesp alleviated the diazinon [53] and aluminum phosphide [54]-induced anemia, leukopenia, and thrombocytopenia. These effects could be attributed to the stimulation of the synthesis and secretion of erythropoietin by Hesp that could result in the rapid synthesis of RBCs [55]. Previously, Mahmoud [52] displayed that Hesp could protect from diabetes-associated anemia by attenuating the pro-inflammatory cytokine production and enhancing the expression of adiponectin, and, consequently, increases RBC production. Overall, the recorded improvement in the hematological parameters associated with the administration of Hesp could be attributed to its antioxidant and anti-inflammatory activities, which enhanced the hematopoietic recovery and modulated the EAC-induced bone marrow suppression [12]. The nephroprotective effect of Hesp was evidenced by the marked decrease in serum urea and creatinine levels, along with the recorded reduction in the MDA level and the increases in the GSH content and CAT activity in renal tissue of EAC-bearing mice following Hesp administration alone or in combination with Cis. Similar to these findings, Sahu et al. [56] suggested the role of Hesp against Cis-induced nephrotoxicity. The microscopic examination supported the biochemical findings, where a marked improvement in the renal histoarchitecture was observed in the renal tissue of EAC-bearing mice treated with Hesp alone or in combination with Cis, along with weak Ki-67 and strong caspase-3 expressions of neoplastic cells infiltrating the renal capsule. These findings partially correlated to previous studies of Donia et al. [16], who suggested that Hesp exerted antitumor properties and possessed an ameliorative effect against doxorubicin cytotoxicity, while enhancing its antitumor effect, attributing this to the ability of Hesp to arrest tumor growth via the apoptotic pathway by downregulating the expression of Bcl-2, an antiapoptotic gene, and upregulating the expression of the Caspase3 and Bax genes, apoptotic genes. Another study explained that the antitumor mechanism of Hesp may be mediated by the induction of apoptosis and prevention of oxidative damage [44].

## 4. Materials and Methods

### 4.1. Chemicals

Cisplatin (Cisplatin**^®^** Mylan, Saint-Priest, France, 50 mg/50 mL vial) was obtained from a local pharmacy, Sadat City, Egypt. Hesperidin (≥80% purity) was purchased from Sigma-Aldrich Chemical Company (St. Louis, MO, USA). Diagnostic kit for serum carcinoembryonic antigen (CEA) level was purchased from Mybiosource Company (San Diego, CA, USA), while those for assaying serum urea and creatinine levels, they were purchased from Spectrum Diagnostic Company (Obour City, Cairo, Egypt), and for assaying renal malondialdehyde (MDA) level, reduced glutathione (GSH) content, and catalase (CAT) activity, they were purchased from Biodiagnostics Company (Dokki, Giza, Egypt). Other utilized chemicals and reagents were of analytical grade and commercially available.

### 4.2. Experimental Animals

This study was ethically approved by the International Animal Care and Use Committee (IACUC), Faculty of Veterinary Medicine, University of Sadat City, Egypt (approval no. VUSC-003-1-21). Seventy adult female Swiss albino mice, weighing 25–30 g, were obtained from the Animal Care Unit of Vacsera Pharmaceutical Company, Giza, Egypt. All animals were housed in polypropylene cages with mesh wire tops at a room with standard conditions (natural daily dark/light cycle; temperature 23 ± 2 °C; humidity 45–50%) and received free access to clean food and tap water during the acclimatization period and throughout the experiment.

### 4.3. Ehrlich Ascites Carcinoma (EAC) Cells and Tumor Inoculation

EAC-bearing mice were obtained from the National Cancer Institute (Cairo University, Egypt). Induction of EAC in the experimental mice was initiated by aspiration of 0.2 mL of ascetic fluid from the EAC-bearing mice, which was diluted with sterile isotonic saline at 1:4 dilution rate. Each mouse was injected intraperitoneally with 2.5 × 10^6^ viable EAC cells/0.2 mL diluted ascetic fluid [57].

### 4.4. Experimental Design

A total of seventy adult female Swiss albino mice was randomly assigned into 7 groups, 10 mice each (Table 5).

Control group: Mice were orally administered distilled water, day by day, till the end of the experiment.

Hesp group: Mice were orally administered Hesp at a dose of 100 mg/kg [58], day by day, till the end of the experiment.

EAC group: Mice were injected (i.p) with 0.2 mL of 2.5 × 10^6^ EAC cells/mouse on day “0” [57].

Hesp-protected (Hesp then EAC) group: Mice were orally administered Hesp (100 mg/kg), day by day, for two weeks before EAC inoculation, and 3 days after EAC inoculation; mice were orally treated with Hesp (100 mg/kg), day by day, till the end of the experiment.

Hesp-treated (EAC then Hesp) group: Mice were orally administered distilled water, day by day, for two weeks before EAC inoculation, and 3 days after EAC inoculation; mice were orally treated with Hesp (100 mg/kg), day by day, till the end of the experiment.

Cis-treated (EAC then Cis) group: Mice were orally administered distilled water, day by day, for two weeks before EAC inoculation, and 3 days after EAC inoculation; mice were treated with a single i.p dose of Cis (5 mg/kg) [49].

Cis+Hesp-treated (EAC then Cis and Hesp) group: Mice were orally administered distilled water, day by day, for two weeks before EAC inoculation, and 3 days after EAC inoculation; mice were treated with a single i.p dose of Cis (5 mg/kg) and Hesp (100 mg/kg) orally, day by day, till the end of the experiment.

### 4.5. Samples Collection and Preparation

By the end of the experimental period (12 days after EAC inoculation), animals were fasted overnight, weighed, and anaesthetized under inhalation anesthesia of isoflurane. Blood samples were collected from the retro-orbital venous plexus on EDTA for hematological assays, while another blood sample was collected without anticoagulant, centrifuged at 3000 rpm to separate serum for further serum biochemical investigations. After sacrificing of mice, the kidneys from each mouse were excised immediately; one kidney was washed in cold normal saline solution, blotted over filter paper and then stored at −80 °C for analyses of oxidant/antioxidant markers, while the other kidney was fixed in 10% neutral-buffered formalin for histopathology and immunohistochemistry investigations.

### 4.6. Investigation of Survival Time

All mice were observed daily for recording of mortalities in each group from day zero till the end of the experiment. The mean survival time (MST) was calculated according to the following equation: MST/day = (First Death + Last Death)/2 [59]. The increase in life span percentage (ILS %) in each group was assessed as follows: ILS % = [(T − C)/C] × 100. T is the number of survival days in the treated group and C is the number of survival days of the control group [60].

### 4.7. Investigation of Tumor Growth

Tumor growth was assessed by measuring changes in the body weight, abdominal circumference, ascitic fluid volume, and the count of viable and nonviable tumor cells [61]. The body weight and abdominal circumference of mice in each group were measured just before EAC inoculation and were observed every day from day zero till the end of the experiment. The ascitic fluid was aspirated from the peritoneal cavity of EAC-bearing mice, using 5 mL disposable syringe and the volume was measured using a graduated centrifuge tube [62]. The aspirated ascitic fluid from each mouse was diluted with trypan blue 1% (9:1) and then incubated for exactly 10 min at 37 °C in water bath; then, within 5 min, the number of viable (unstained) and nonviable (stained) cells were counted using a Neubauer hemocytometer [63]. The viable cells were counted by the following formula: Viable cells count = (Number of cells × Dilution factor)/(Area × Thickness of liquid film) [64].

### 4.8. Assessment of Serum Carcinoembryonic Antigen Level

Serum level of CEA (CAT.NO. CSB-E13925m) was estimated by quantitative sandwich immunoassay technique according to the method described by Lewis et al. [65], using specific monoclonal antibody for mouse CEA and following the manufacturer’s instructions.

### 4.9. Investigation of Hematological Indices

The evaluated hematological parameters in this study included the estimation of red blood cell counts (RBCs), hemoglobin concentration (Hb), packed cell volume (PCV), mean corpuscular volume (MCV), mean corpuscular hemoglobin (MCH), mean corpuscular hemoglobin concentration (MCHC), and total leukocytic (TWBCs) and differential leukocytic counts, as well as platelets count. All hematological parameters were performed using a BeneSphera brand 3-part differential veterinary hematology analyzer H23 (Avantor Performance Materials, Inc, Deventer,The Netherlands; model: H23vet. Serial no: 931716004) according to the procedures adopted by Grindem [66].

### 4.10. Assessment of Serum Renal Function

Serum urea (CAT.NO.318001) and creatinine (CAT.NO.235001) levels were measured according to the methods adopted by Tietz [67] and Tietz [68], respectively, using commercial kits according to the manufacturer’s instructions.

### 4.11. Assessment of Renal Oxidant/Antioxidant Biomarkers

Renal tissue contents of MDA (CAT.NO.MD 2529) and GSH (CAT.NO. GR 2511) and CAT activity (CA 25 17) were estimated using colorimetric method according to Ohkawa et al. [69], Beutler et al. [70], and Aebi [71], respectively.

### 4.12. Histopathological Examination of Renal Tissue

The formalin-fixed kidney tissue specimens were trimmed, processed for paraffin sections (5 µm thicknesses), stained with hematoxylin and eosin for histopathological examination according to Bancroft and Gamble [72]. Semiquantitation for renal lesions, including vascular, inflammatory, degenerative, and neoplastic changes, were scored according to the degree of severity as no changes (0), mild (1), moderate (2), and severe (3) changes, and the grading was determined by percentage as follows: <30% changes (mild change), 30–50% (moderate change), and >50% (severe change) [73].

### 4.13. Immunohistochemical Examination of Renal Tissue

Immunohistochemical analysis was carried out following the methods described by El-Maksoud et al. [74]. Tissue sections from kidneys were deparaffinized in xylene and rehydrated in graded alcohol. Hydrogen Peroxide Block (Thermo scientific, Waltham, MA, USA) was added to block the endogenous peroxidase activity. Antigen retrieval was performed by pretreated tissue sections with 10 mM citrate in a microwave oven for 10 min. Sections were incubated for 2 h with one of the following primary monoclonal antibodies for Ki-67 (1:250) and caspase-3 (1:100) (Dako Corp, Carpinteria, CA, USA). The sections were rinsed with PBS then incubated with goat anti-rabbit IgG H&L (HRP) (ab205718; Abcam, Cambridge, UK) for 10 min. The sections were rinsed again with PBS. Finally, sections were incubated with 3, 3′-diaminobenzidine tetrahydrochloride (DAB, Sigma, VWR International GmbH, Graumanngasse, Vienna). The slides were counterstained with hematoxylin then mounted. Primary antibodies were replaced by PBS for negative controls.

The quantitative immunoreactivity of Ki-67 and caspase-3 was evaluated in neoplastic cells in each group according to Azouz and Korany [75]; five kidney sections were examined. Immuno-reactivity was analyzed in 10 microscopical fields per each section under high-power microscopic field (×400). The percentage of positively stained cells (%) was estimated by color deconvolution image J 1.52 p software (Wayne Rasband, National Institutes of Health (USA)).

### 4.14. Statistical Analysis

Data were expressed as mean values ± SE. The statistical analyses were performed by using one-way ANOVA followed by Duncan’s post hoc using SPSS**^®^** (Statistical Package for Social Sciences) Version 26, IBM Inc. (Chicago, IL, USA) to determine the statistically significant differences among the experimental groups. The differences were considered statistically significant at *p* < 0.05.

## 5. Conclusions

Our findings provided evidence for the antitumor efficacy of Hesp, mediated by the induction of apoptosis and prevention of oxidative damage, and its ability to alleviate the hematological and renal adverse effects in EAC-bearing mice. In addition, Hesp promoted the antitumor potency of Cis, while minimizing its adverse side effects. Therefore, the overall obtained findings may be of crucial value in presenting Hesp as a new safe and efficient strategy for cancer treatment and for ameliorating the cytotoxicity associated with chemotherapeutic drugs.

## Figures and Tables

**Table 1:** Effect of hesperidin and/or cisplatin on survival time, tumor growth response parameters, and serum tumor marker of EAC-bearing mice.

Parameters	Experimental Groups						
	Control	Hesp	EAC	Hesp-Protected	Hesp-Treated	Cis-Treated	Cis+Hesp-Treated
*Survival time*							
MST (days)	-	-	17	31	19.5	24.5	30.5
ILS (%)	-	-	0	82.35	14.7	44.12	79.41
*Tumor growth response*							
B.wt. (g)	31.60 ± 0.34 ^d^	30.60 ± 0.26 ^d^	45.50 ± 0.54 ^a^	31.70 ± 0.36 ^d^	39.25 ± 0.34 ^b^	34.90 ± 0.50 ^c^	31.75 ± 0.43 ^d^
AC (mm)	7.85 ± 0.09 ^d^	8.05 ± 0.08 ^d^	11.70 ± 0.15 ^a^	8.18 ± 0.10 ^d^	11.10 ± 0.18 ^b^	9.60 ± 0.26 ^c^	8.30 ± 0.12 ^d^
AFV (mL)	-	-	9.87 ± 0.37 ^a^	1.09 ± 0.04 ^e^	6.63 ± 0.21 ^b^	3.90 ± 0.11 ^c^	2.81 ± 0.09 ^d^
VCC(×10^6^/mL)	-	-	21.27 ± 0.75 ^a^	4.30 ± 0.40 ^d^	12.39 ± 0.53 ^b^	6.90 ± 0.57 ^c^	5.30 ± 0.39 ^d^
NCC(×10^6^/mL)	-	-	0.95 ± 0.03 ^c^	2.40 ± 0.20 ^a^	1.46 ± 0.15 ^b^	1.94 ± 0.14 ^a^	2.23 ± 0.19 ^a^
*Tumor marker*							
CEA (ng/mL)	0.34 ± 0.03 ^c^	0.33 ± 0.03 ^c^	1.18 ± 0.08 ^a^	0.45 ± 0.04 ^c^	0.94 ± 0.05 ^b^	0.81 ± 0.05 ^b^	0.49 ± 0.04 ^c^

Values are means ± SE. Different letters (a, b, c, d, e) in the same row indicate significant differences at *p* < 0.05. EAC: Ehrlich ascites carcinoma; Hesp: hesperidin; Cis: cisplatin; MST: mean survival time; ILS: increased life span percentage; B.wt.: body weight; AC: abdominal circumference; AFV: ascitic fluid volume; VCC: viable EAC cells count; NCC: nonviable EAC cells count; CEA: carcinoembryonic antigen.

**Table 2:** Effect of hesperidin and/or cisplatin on erythrogram parameters of EAC-bearing mice.

Parameters	Experimental Groups						
	Control	Hesp	EAC	Hesp-Protected	Hesp-Treated	Cis-Treated	Cis+Hesp-Treated
RBCs (×10^6^)	8.84 ± 0.35 ^a^	8.77 ± 0.29 ^a^	6.15 ± 0.70 ^b^	8.58 ± 0.34 ^a^	6.30 ± 0.56 ^b^	5.80 ± 0.29 ^b^	8.23 ± 0.28 ^a^
Hb (g/dL)	13.16 ± 0.35 ^a^	12.82 ± 0.43 ^a^	10.02 ± 0.66 ^b^	12.68 ± 0.29 ^a^	10.32 ± 0.42 ^b^	9.03 ± 0.45 ^b^	11.80 ± 0.38 ^a^
PCV (%)	41.12 ± 0.65 ^a^	40.50 ± 0.84 ^a^	35.40 ± 0.93 ^b^	39.00 ± 0.71 ^a^	35.66 ± 0.97 ^b^	30.80 ±0.92 ^c^	38.74 ± 0.70 ^a^
MCV (fl)	46.76 ± 1.40 ^b^	46.36 ± 1.54 ^b^	57.56 ± 1.90 ^a^	45.64 ± 1.41 ^b^	56.60 ± 1.78 ^a^	53.43 ± 1.60 ^ab^	47.19 ± 1.16 ^b^
MCH (pg)	14.95 ± 0.56 ^a^	14.64 ± 0.37 ^a^	16.38 ± 0.90 ^a^	14.87 ± 0.69 ^a^	16.38 ± 1.20 ^a^	15.85 ± 1.48 ^a^	14.34 ± 0.27 ^a^
MCHC (g/dL)	32.00 ± 0.37 ^a^	31.75 ± 1.53 ^a^	28.38 ± 1.50 ^a^	32.53 ± 0.68 ^a^	28.96 ± 1.54 ^a^	29.51 ± 1.90 ^a^	30.44 ± 0.65 ^a^

Values are means ± SE (*n* = 7). Different letters (a, b, c) in the same row indicate significant differences at *p* < 0.05. EAC: Ehrlich ascites carcinoma; Hesp: hesperidin; Cis: cisplatin; RBCs: red blood cells count; Hb: hemoglobin concentration; PCV: packed cell volume; MCV: mean corpuscular volume; MCH: mean corpuscular hemoglobin; MCHC: mean corpuscular hemoglobin concentration.

**Table 3:** Effect of hesperidin and/or cisplatin on leukogram and blood platelets of EAC-bearing mice.

Parameters(×10^3^/µL)	Experimental Groups						
	Control	Hesp	EAC	Hesp-Protected	Hesp-Treated	Cis-Treated	Cis+Hesp-Treated
TWBCs	8.70 ± 0.82 ^b^	8.81 ± 0.67 ^b^	12.48 ± 0.40 ^a^	8.84 ± 0.58 ^b^	11.41 ± 0.86 ^a^	6.76 ± 0.60 ^c^	9.02 ± 0.53 ^b^
Granulocytes	0.82 ± 0.07 ^c^	0.86 ± 0.05 ^c^	2.62 ± 0.28 ^a^	0.93 ± 0.06 ^c^	1.94 ± 0.18 ^b^	1.12 ± 0.07 ^c^	0.97 ± 0.05 ^c^
Lymphocytes	6.89 ± 0.61 ^a^	6.79 ± 0.75 ^a^	6.34 ± 0.34 ^ab^	6.74 ± 0.62 ^a^	6.38 ± 0.48 ^ab^	4.89 ± 0.37 ^b^	6.68 ± 0.38 ^a^
Monocytes	0.99 ± 0.06 ^b^	1.16 ± 0.10 ^b^	3.52 ± 0.31 ^a^	1.17 ± 0.11 ^b^	3.09 ± 0.45 ^a^	0.75 ± 0.09 ^b^	1.37 ± 0.17 ^b^
Platelets	688.8 ± 20.0 ^a^	660.0 ± 22.2 ^a^	552.2 ± 18.3 ^b^	656.4 ± 24.8 ^a^	550.0 ± 22.8 ^b^	446.8 ± 19.9 ^c^	630.9 ± 21.1 ^a^

Values are means ± SE (*n* = 7). Different letters (a, b, c) in the same row indicate significant differences at *p* < 0.05. EAC: Ehrlich ascites carcinoma; Hesp: hesperidin; Cis: cisplatin; TWBCs: total white blood cells.

**Table 4:** Scoring of histopathological alterations in the kidneys of the different treated groups.

Lesions	Experimental Groups						
	Control	Hesp	EAC	Hesp-Protected	Hesp-Treated	Cis-Treated	Cis+Hesp-Treated
Congestion of glomerular tuft	0	0	3	1	3	3	1
Congestion of interstitial blood vessels	0	0	3	1	3	3	1
Mononuclear inflammatory cells infiltration in interstitial tissue	0	0	3	1	3	3	2
Renal tubular vacuolar degeneration	0	0	3	1	3	3	1
Renal tubular necrosis	0	0	3	0	3	3	0
Tubular casts	0	0	2	0	2	3	1
Neoplastic cells infiltrating capsule and blood vessels	0	0	3	1	3	1	1

The score system was designed such as that score of 0 means absence of lesions in all mice of the group (*n* = 5), score of 1 means (<30%), score of 2 means (<30–50%), and score of 3 means (>50%). EAC: Ehrlich ascites carcinoma; Hesp: hesperidin; Cis: cisplatin.

**Table 5:** The experimental design and animal grouping.

Groups	Experimental Design			
	Preinoculation (1st–15th Day)	Inoculation Day 0 (16th Day)	Post-Inoculation	
			3 Days after Inoculation (19th Day)	12 Days after Inoculation (28th Days)
**Control**	Distilled water, orally, day by day			Day of scarification
**Hesp**	Hesp (100 mg/kg) orally day by day dissolved in distilled water			
**EAC**	-	0.2 mL containing 2.5 × 10^6^ EAC cells/mouse, i.p	-	
**Hesp-protected** (Hesp then EAC)	Hesp (100 mg/kg orally day by)		Hesp (100 mg/kg, orally, day by day)	
**Hesp-treated** (EAC then Hesp)	Distilled water orally day by day		Hesp (100 mg/kg, orally, day by day)	
**Cis-treated** (EAC then Cis)	Distilled water orally day by day		Cis (single dose of 5 mg/kg i.p)	
**Cis+Hesp-treated** (EAC then Cis and Hesp)	Distilled water orally day by day		Cis (Single dose of 5 mg/kg i.p) and Hesp (100 mg/kg, orally, day by day)	

EAC: Ehrlich ascites carcinoma; Hesp: hesperidin; Cis: cisplatin.

## Data Availability

Data is contained within the article.
